# Muscle weakness, functional capacities and recovery for COVID-19 ICU survivors

**DOI:** 10.1186/s12871-021-01274-0

**Published:** 2021-03-02

**Authors:** Clément Medrinal, Guillaume Prieur, Tristan Bonnevie, Francis-Edouard Gravier, Denys Mayard, Emmanuelle Desmalles, Pauline Smondack, Bouchra Lamia, Yann Combret, Guillaume Fossat

**Affiliations:** 1 Normandie Univ, UNIROUEN, EA3830-GRHV, 76 000 Rouen, France; 2 Institute for Research and Innovation in Biomedicine (IRIB), 76 000 Rouen, France; 3 Intensive Care Unit Department, Groupe Hospitalier du Havre, Avenue Pierre Mendes France, 76290 Montivilliers, France; 4 IFMK Saint Michel, 75015 Paris, France; 5 Research and Clinical Experimentation Institute (IREC), Pulmonology, ORL and Dermatology, Louvain Catholic University, 1200 Brussels, Belgium; 6 ADIR Association, Bois-Guillaume, France; 7 Service de Médecine Intensive Réanimation, Centre Hospitalier Régional d’Orléans, Orléans, France; 8 Pulmonology Department, Groupe Hospitalier du Havre, Avenue Pierre Mendes France, 76290 Montivilliers, France; 9 Pulmonology, Respiratory Department, Rouen University Hospital, Rouen, France

**Keywords:** COVID-19, Intensive care unit, Mechanical ventilation, Muscle weakness, Physiotherapy

## Abstract

**Background:**

Few studies have evaluated muscle strength in COVID-19 ICU survivors. We aimed to report the incidence of limb and respiratory muscle weakness in COVID-19 ICU survivors.

**Method:**

We performed a cross sectional study in two ICU tertiary Hospital Settings. COVID-19 ICU survivors were screened and respiratory and limb muscle strength were measured at the time of extubation. An ICU mobility scale was performed at ICU discharge and walking capacity was self-evaluated by patients 30 days after weaning from mechanical ventilation.

**Results:**

Twenty-three patients were included. Sixteen (69%) had limb muscle weakness and 6 (26%) had overlap limb and respiratory muscle weakness. Amount of physiotherapy was not associated with muscle strength. 44% of patients with limb weakness were unable to walk 100 m 30 days after weaning.

**Conclusion:**

The large majority of COVID-19 ICU survivors developed ICU acquired limb muscle weakness. 44% of patients with limb weakness still had severely limited function one-month post weaning.

## Background

The rate of intensive care unit admissions due to coronavirus (COVID-19) infections was very high and a large proportion of these patients required invasive ventilation [1]. Evidence from studies carried out worldwide shows that patients who undergo invasive ventilation in ICU have a high risk of developing respiratory and limb muscle weakness (50% prevalence) [2]. It is therefore reasonable to expect that a large proportion of COVID-19 ICU survivors will develop such weakness. Guidelines from an international team of expert physiotherapist researchers and clinicians recommend early physiotherapy in the ICU to prevent ICU-acquired weakness [3].

Although a large number of studies into various aspects of COVID-19 have been published [4], to our knowledge, few studies have evaluated muscle strength in COVID-19 ICU survivors [5]. There is also a paucity of data relating strength to physiotherapy interventions carried out during the period of mechanical ventilation (MV). The primary aim of this study was to report the prevalence of limb and respiratory muscle weakness in COVID-19 ICU survivors. The secondary aims were to analyse variables associated with muscle weakness.

## Method

We conducted a retrospective, observational study in two centres, each with a 30 bed ICU. In accordance with current French legislation, written informed consent was unnecessary for this study. Data were collected and treated in accordance with the French Institutional Review Board (CNIL ID-number n°2,220,110) and in conformity with the Declaration of Helsinki. Patients with laboratory confirmed COVID-19 infection who were intubated for at least 24 h and were hospitalized between the 16th of March and the 15th of May 2020 were included.

In both centres, all patients received physiotherapy. In center 1: rehabilitation started when administration of neuromuscular blockers ceased. Quadriceps electrical muscle stimulation if the patient could not respond to simple commands. Active mobilization was started in bed once the patient could follow commands. Sitting over the edge of the bed was initiated when the patient’s haemodynamic condition was stable. Inspiratory muscle training (IMT) was initiated when the patient had shifted to a pressure support ventilation mode.

In center 2: rehabilitation started with passive mobilisation when the patient was still under neuromuscular blockers. Active mobilization was started in bed once the patient could follow commands. Sitting over the edge of the bed was initiated when the patient’s haemodynamic condition was stable. Electrical muscle stimulation and inspiratory muscle training were not performed.

Respiratory and limb muscle strength were measured at the time of extubation if the patient was sufficiently alert and cooperative to respond to instructions. Three measurements of maximal inspiratory pressure (MIP) were carried out and the best result was used in the analysis. Respiratory weakness was defined as an MIP ≤ 30cmH2O [6]. Limb muscle strength was evaluated using the MRC scale with weakness defined as a score ≤ 48/60. Patients’ functional status on discharge from ICU was evaluated with the ICU mobility scale (IMS).

Patients were contacted by telephone 30 days after mechanical ventilation weaning and asked if they could walk more than 100 m.

### Statistical analysis

Descriptive statistics are reported as counts and percentages for categorical data, and means and standard deviations or medians and interquartile ranges, according to the distribution, for continuous variables.

Patients’ baseline characteristics were compared between groups with limb weakness and no weakness using a Student *t* test or a Wilcoxon-Mann-Whitney test, as appropriate, for continuous variables and using a Chi-square test or Fisher exact test, as appropriate, for categorical variables. Significative variables were included in a multiple regression analysis model. Statistical analyses were performed using GraphPad Prism 5 (GraphPad Software, Inc., La Jolla, CA, USA). A two-tailed *p* value of 0.05 was considered significant for all analyses.

## Results

During the study period, 89 patients with confirmed COVID-19 were admitted to ICU in both centres, and 65 required invasive MV. Twenty-four patients died before weaning and the evaluation could not be carried out for 18 patients during the weaning process because of neurological disorders, agitation or lack of staff to perform the evaluations, thus data from 23 patients were analysed.

Patient characteristics are presented in Table 1.

**Table 1:** Patients characteristics

Characteristics	All patients *N* = 23	Limbs weakness *N* = 16	No Limbs weakness *N* = 7	*P*-Value
Gender F/M, n	6/17	3/13	3/4	0.31
Age	64.6 ± 9.6	65.8 ± 11	62.4 ± 8	0.47
Weight (Kg)	85.2 ± 12.3	86.2 ± 13.2	82.9 ± 10.5	0.25
Body Mass Index (Kg/m^2^)	29.1 ± 3.5	28.9 ± 3.3	29.4 ± 4.4	0.74
SAPS II at ICU admission	39.7 ± 14.9	38.1 ± 15.6	43.3 ± 13.5	0.45
**Co-morbidity**				
Chronic Pulmonary Disease, n (%)	9 (39)	6 (37)	3 (43)	1
Chronic Cardiac Insufficiency, n (%)	6 (23)	5 (31)	1 (14)	0.11
Obesity, n (%)	9 (39)	6 (37)	3 (43)	0.49
Chronic Kidney Disease, n (%)	0 (0)	0 (0)	0 (0)	–
Diabetes mellitus, n (%)	8 (35)	4 (25)	4 (57)	0.18
Hypertension, n (%)	11 (48)	7 (44)	4 (57)	0.66
**Between admission and awakening**				
Septic shock, n (%)	13 (56)	11 (69)	2 (46)	0.16
Use of catecholamines, n (%)	16 (69)	14 (87)	2 (46)	0.01
No. of days of neuromuscular blockers	4.9 ± 3.8	5.6 ± 4	3 ± 2.3	0.11
No. of days on prone position	1 (2)	3.2 ± 2.8	0.8 ± 1.6	0.04
Use of corticosteroid, n (%)	2 (12)	1 (6)	1 (14)	0.5
Ventilator use (days)	15.3 ± 10.3	18.5 ± 10.4	8.2 ± 5.5	0.02
AC mode ventilation use	8.9 ± 6.2	10.7 ± 6.3	4.8 ± 3.6	0.03
PS mode ventilation use	5.8 ± 4.4	6.9 ± 4.7	3.4 ± 2.4	0.08
No of physiotherapy sessions during MV	9 ± 8.3	12 ± 8.2	3 ± 4.3	0.01
ICU length of stay	22.2 ± 12.3	25.9 ± 12.3	13.6 ± 7.1	0.02
**Muscle and physical function**				
MRC score	41 ± 14	34 ± 11.2	56.6 ± 3.8	< 0.001
MIP value	40 ± 11	37.7 ± 11.8	46.1 ± 6.3	0.11
Peak expiratory cough flow	94.2 ± 34.8	95.4 ± 39.7	91 ± 18.5	0.79
ICU mobility scale at ICU discharge	5.4 ± 2.1	4.3 ± 1.5	8 ± 0.6	< 0.001
Walk less than 100 m at 1 month	7 (31)	7 (44)	0 (0)	0.06
Rehabilitation access after ICU	10 (43)	9 (56)	1 (14)	0.08

Variables are expressed in mean ± SD or n with percentage. Abbreviations: *SAPS* Simplified Acute Physiology Score, *ICU* Intensive Care Unit, *No* Number, *AC* Assit-Controlled, *PS* Pressure Support, *MRC* Medical Research Council, *MIP* Maximal Inspiratory Pressure

**Fig. 1:**
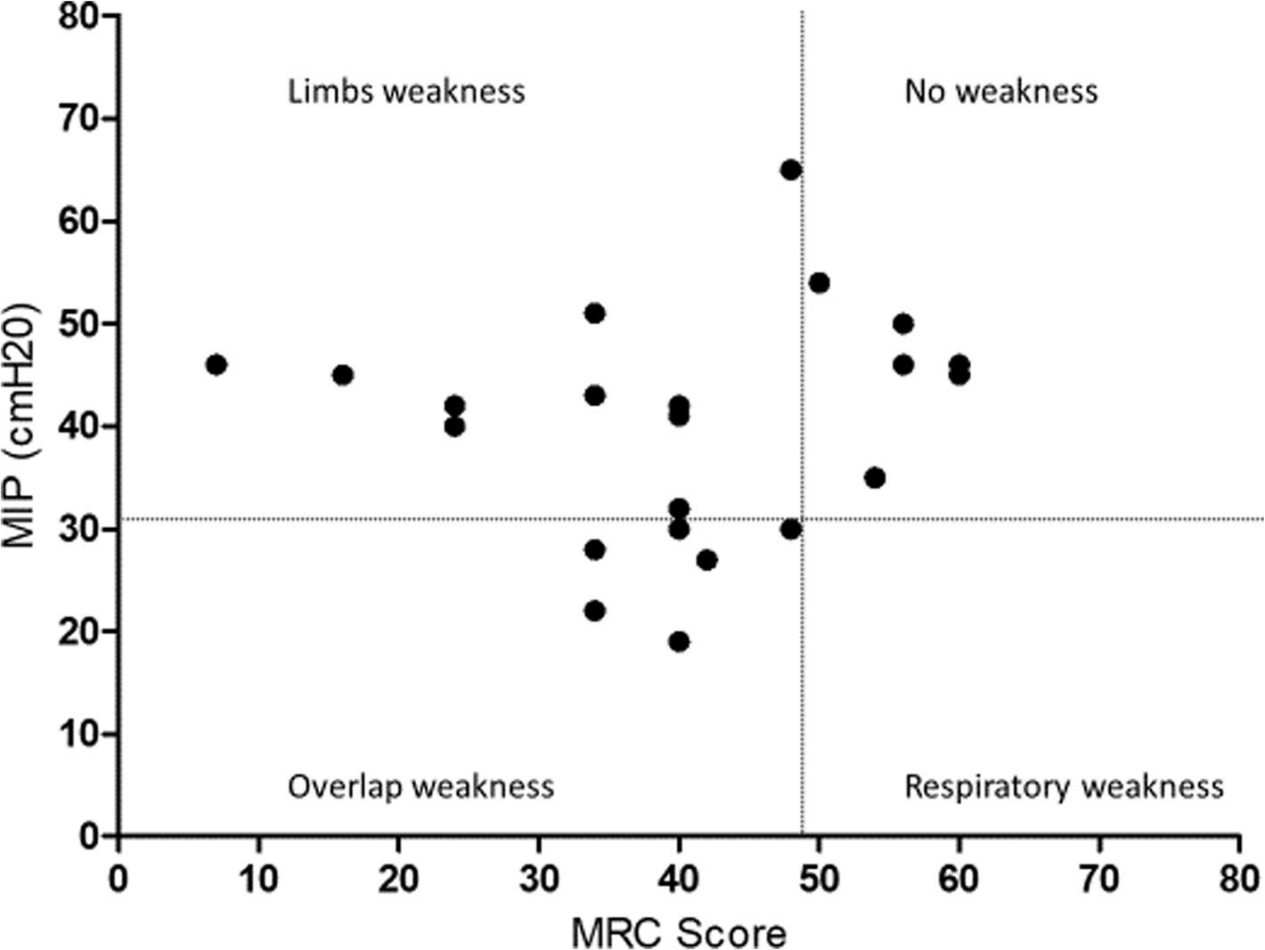
Individual values of the MRC-Score according to the MIP

Figure 1 shows individual classifications of the patient according to their strength. Of 23 patients, 16 (69%) had limb muscle weakness and 6/23 (26%) had overlap (limb and respiratory muscle weakness) weakness [7]. Limb muscle weakness was significantly associated with the number of days spent in a prone position, the use of catecholamines, and the number of days under MV (Table 1), particularly with the use of assist-control ventilation (10.6 ± 6.3 vs. 4.8 ± 3.6 days; *p* = 0.03). Table 2 shows the physiotherapy intervention performed during mechanical ventilation. Passive range of motion, in-bed strengthening, neuromuscular electrical stimulation (NMES), inspiratory muscle training and out-of-bed verticalization was the commons physiotherapy intervention. The number of sessions of physiotherapy was not associated with higher muscle strength. Some inspiratory muscle training sessions induced adverse events with rapid resolution (bradycardia less than 50 bpm). Multiple regression analysis showed that muscle weakness was only independently associated with the number of days under MV (*p* = 0.005) (See Table 3). There was a statistically significant difference between the ICU mobility scale scores of the two groups. Patients with muscle weakness had lower IMS scores (4.3 ± 1.5 vs. 8 ± 0.6; *p* < 0.001).

**Table 2:** Description of physiotherapy intervention

Physiotherapy intervention	All patients *N* = 23	Limbs weakness *N* = 16	No Limbs weakness *N* = 7	*P*-Value
Passive Range of motion; n	132	120	12	0.07
Adverse events; n (%)	0 (0)	0 (0)	0 (0)	
Bed ridden strengthening; n	8	6	2	0.7
Adverse events; n (%)	0 (0)	0 (0)	0 (0)	
Neuromuscular electrical stimulation; n	17	15	2	0.78
Adverse events; n (%)	0 (0)	0 (0)	0 (0)	
Inspiratory muscles training; n	30	23	7	0.6
Adverse events; n (%)	5 (16)	4 (17)	1 (14)	
Verticalization out of bed; n	28	28	0	0.049
Adverse events; n (%)	0 (0)	0 (0)	0 (0)	

Variables was expressed as counts and percentage

**Table 3:** Multiple regression analysis

	Estimate	95% Confidence interval	*p*-value
Mechanical ventilation (days)	−0.87	−1.45 to −0.29	0.0055
Prone position (days)	0.02	−2.28 to 2.32	0.98
Catecholamine (use)	−5.63	−16.7 to 5.4	0.29

Briefly, patients in the Limb weakness group acquired the standing position while the patients in the no weakness group could walk at least 5 m away from the bed/chair, assisted by 1 person.

Of the patients with limb muscle weakness (*n* = 16), 7 (44%) were unable to walk 100 m 30 days after weaning, however 6 of them could walk shorter distances. All the patients who did not have limb muscle weakness were able to walk 100 m or more.

## Discussion

The results of this study showed that a high proportion of COVID-19 survivors developed ICU acquired muscle weakness, despite early physiotherapy, and 44% were unable to walk 100 m 30 days post discharge.

Despite receiving early, evidence-based physiotherapy with therapists who were all experienced in ICU physiotherapy [8, 9], a high proportion of patients developed muscle weakness. The rate of limb muscle weakness found in this cohort of patients was also higher than in patients without COVID-19 [10], which could be due to the longer duration of MV. The higher number of physiotherapy sessions in the Limb weakness group is also due to the longer duration of mechanical ventilation in that group.

The incidence of ICU muscle weakness in this cohort was similar to that found by Van Aerde et al. [5]. These findings highlight the necessity to try to (1) decrease the use of invasive mechanical ventilation, (2) increase early rehabilitation intensity for the prevention of ICU acquired weakness in patients who undergo long periods of MV, (3) anticipate the need for rehabilitation after ICU, and (4) enhance post-ICU follow-up to monitor weakness and its long-term impact [11].. Despite our results, it is possible that early physiotherapy intervention decreased the severity of weakness. Post-discharge, patients who required further physiotherapy continued with outpatient (or home) physiotherapy, and it is encouraging to note that only one patient was unable to walk 30 days later, suggesting a potential for recovery of walking in COVID-19 ICU survivors.

Surprisingly, the rate of patients with respiratory muscle weakness was very low and was not associated with any of the other variables analysed. This could be related to the small size of the cohort and the fact that pressure support mode was used for around 40% of MV time. The recruitment of the respiratory muscles for this duration is known to reduce the risk of respiratory muscle weakness [12].

### Limitations

Our study design and the low sample size induced some limitations. We couldn’t report precisely the time return at a good functional status and the one-month functional evaluation lacked specificity. Firstly, we did not evaluate upper-limb function. Secondly, the evaluation is not a validated functional test such as the six-minute walk test or the one-minute sit to stand test. In view of the health crisis, it was not possible for patients to return to hospital for follow-up evaluations. We therefore chose this simple self-evaluation and used the distance proposed in the MMRC dyspnoea scale that indicates that patients who walk less than 100 m are unlikely to go out of their homes and are severely limited in their daily activities [13]. Further work is necessary to evaluate the functional consequences in the longer-term using more specific and complete measures.

Finally, in this study, we did not evaluate chest physiotherapy techniques to improve respiratory function in patients with Covid-19 [14, 15]. However, it is important to specify the importance of techniques that can improve respiratory function, reduce the risk of failure to wean from ventilation and to optimise the return to functional independence [16, 17].

## Conclusion

In conclusion, we found that a high majority of COVID-19 ICU survivors developed ICU acquired limb muscle weakness due to the long duration of MV. Early physiotherapy was not sufficient to prevent this from occurring and, in this small study, 44% of patients with limb weakness still had severely limited function one-month post discharge. However, the majority of patients were in the process of recovering function.

## Data Availability

Data were available by contacting corresponding author medrinal.clement.mk@gmail.com
